# Hemojuvelin-Associated Juvenile Hemochromatosis: Fulminant Heart Failure, Complete Heart Block, and Ventricular Arrhythmias

**DOI:** 10.1016/j.jaccas.2026.107948

**Published:** 2026-04-17

**Authors:** Andrew Costa, Abdul Zia, Samantha Weller, Katherine Lutz, Ingy Mahana, Kristina Wakeman, Luke Masha

**Affiliations:** a Department of Internal Medicine, Oregon Health and Science University, Portland, Oregon, USA; b Knight Cardiovascular Institute, Oregon Health and Science University, Portland, Oregon, USA; c Department of Pathology and Laboratory Medicine, Oregon Health and Science University, Portland, Oregon, USA

**Keywords:** cardiac transplant, conduction disease, endomyocardial biopsy, iron overload cardiomyopathy, myocardial iron deposition

## Abstract

**Background:**

Hemojuvelin (*HJV*)-associated juvenile hemochromatosis is an uncommon subtype of hereditary hemochromatosis that often presents before age 30, with significant cardiac complications.

**Case Summary:**

A 28-year-old woman with hypogonadotropic hypogonadism presented with cardiac arrest. Subsequent evaluation revealed biventricular dysfunction, ventricular fibrillation, and complete heart block. Endomyocardial biopsy demonstrated extensive iron deposition, with subsequent genetic confirmation of *HJV* mutations.

**Discussion:**

*HJV* mutation results in a profound elevation of serum iron that preferentially deposits in cardiac tissue owing to high expression of L-type calcium channels. The underlying pathophysiology enables unregulated entry of iron into myocardium, resulting in accelerated development of cardiomyopathy and associated conduction abnormalities.

**Take-Home Message:**

*HJV*-associated juvenile hemochromatosis should be considered in younger adults presenting with idiopathic cardiomyopathy, ventricular arrhythmias, or complete heart block, as timely diagnosis and treatment are essential to prevent devastating cardiovascular outcomes.

## Visual Summary

**Figure:**
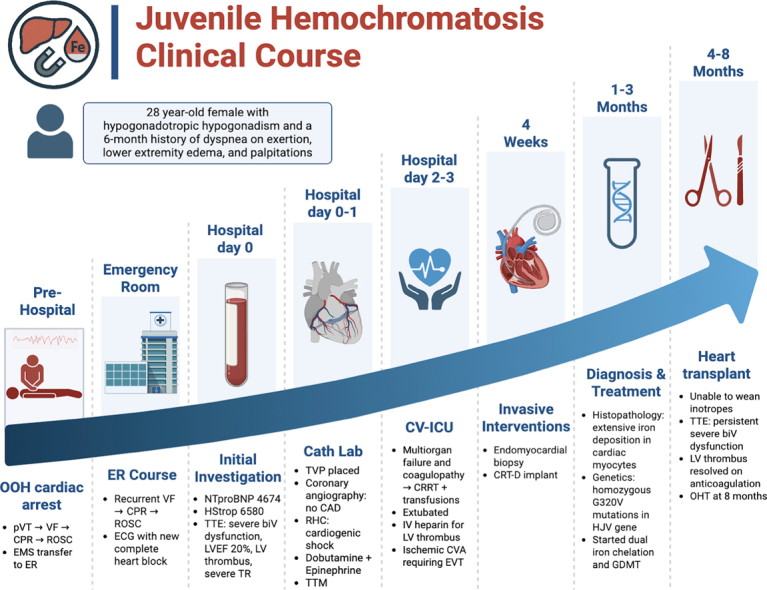

**Visual Summary:**
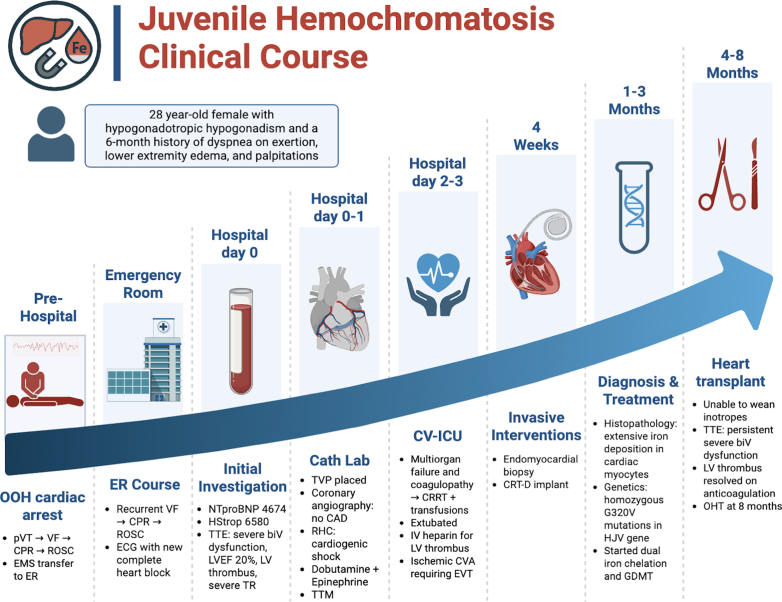
Juvenile Hemochromatosis: Clinical Course Image created in BioRender (Weller S, 2025, https://BioRender.com/k2dt74x). biV = biventricular; CAD = coronary artery disease; CPR = cardiopulmonary resuscitation; CRRT = continuous renal replacement therapy; CRT-D = cardiac resynchronization therapy with defibrillator; CVA = cerebrovascular accident; ECG = electrocardiogram; EMS = emergency medical services; ER = emergency room; EVT = endovascular thrombectomy; GDMT = guideline-directed medical therapy; HStrop = high-sensitivity troponin; IV = intravenous; LV = left ventricle; LVEF = left ventricular ejection fraction; NTproBNP = N-terminal pro-B-type natriuretic peptide; OHT = orthotopic heart transplant; OOH = out-of-hospital; pVT = pulseless ventricular tachycardia; RHC = right heart catheterization; ROSC = return of spontaneous circulation; TR = tricuspid regurgitation; TTE = transthoracic echocardiography; TTM = targeted temperature management; TVP = transvenous pacemaker; VF = ventricular fibrillation.

## History of Presentation

**Figure 1:**
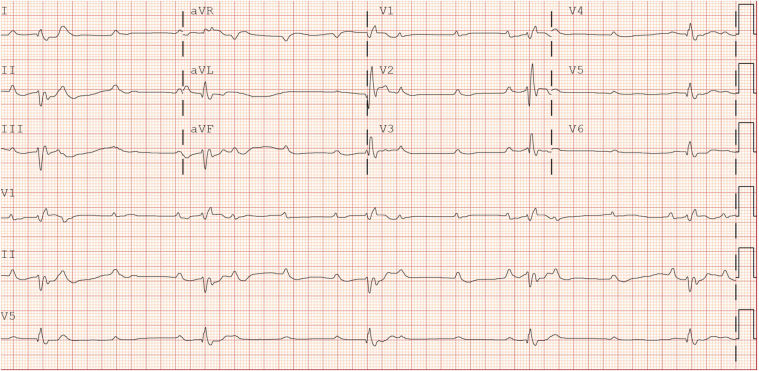
Initial Electrocardiogram With Complete Heart Block Electrocardiogram at presentation in the emergency department demonstrating normal sinus rhythm, complete heart block, left anterior fascicular block, and right bundle branch block.

A 28-year-old woman with a history of idiopathic hypogonadotropic hypogonadism presented to the emergency department after an out-of-hospital cardiac arrest. The initial rhythm was ventricular tachycardia/fibrillation. Advanced cardiac life support was initiated in the field with multiple defibrillations, resulting in return of spontaneous circulation. She was intubated for acute hypoxic respiratory failure. A 12-lead electrocardiogram obtained postresuscitation (Figure 1) demonstrated new-onset complete heart block (CHB). She remained profoundly bradycardic and hypotensive. Physical examination revealed an appearance much younger than stated age, tanned complexion, low body mass index, cyanotic extremities, lower extremity pitting edema, jugular venous distention, and diffuse bilateral crackles on lung auscultation. Hepatosplenomegaly was absent.

**Take-Home Messages**

• *HJV*-associated juvenile hemochromatosis is a rare but important subtype of hereditary hemochromatosis that should be considered in younger adults presenting with idiopathic cardiomyopathy.
• Early recognition of cardiovascular manifestations, proper interpretation of iron studies, and timely initiation of therapy is essential in order to mitigate progression of disease and prevent the devastating cardiovascular outcomes associated with this rare genetic disorder.

## Past Medical History

The patient's medical history consisted of amenorrhea and idiopathic hypogonadotropic hypogonadism. She underwent prior gynecological consultation for these issues, and brain magnetic resonance imaging was normal. Over the preceding 6 months, she had undergone evaluation for progressive dyspnea on exertion, lower extremity edema, and palpitations. There was no personal or family history of deep vein thrombosis, pulmonary embolism, coagulopathy, or heart failure.

## Differential Diagnosis

Massive pulmonary embolism, acute coronary syndrome, fulminant myocarditis, etiologies of infiltrative and arrhythmogenic cardiomyopathies, and congenital heart disease were all considered.

## Investigations

**Figure 2:**
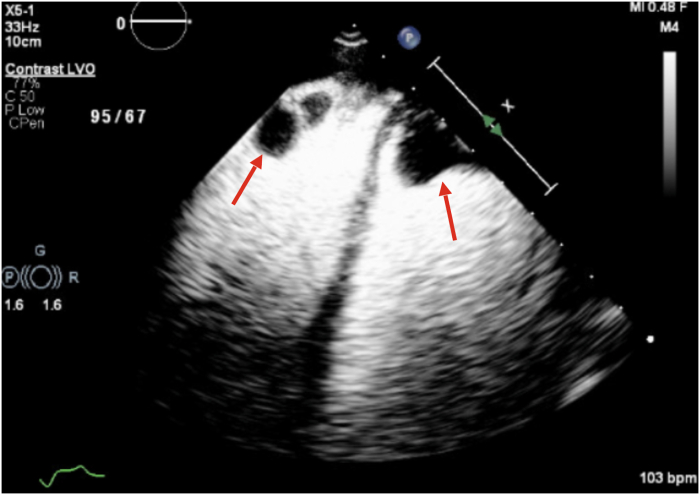
Initial Transthoracic Echocardiogram With Contrast Demonstrating Right and Left Ventricular Thrombi (Arrows)

**Figure 3:**
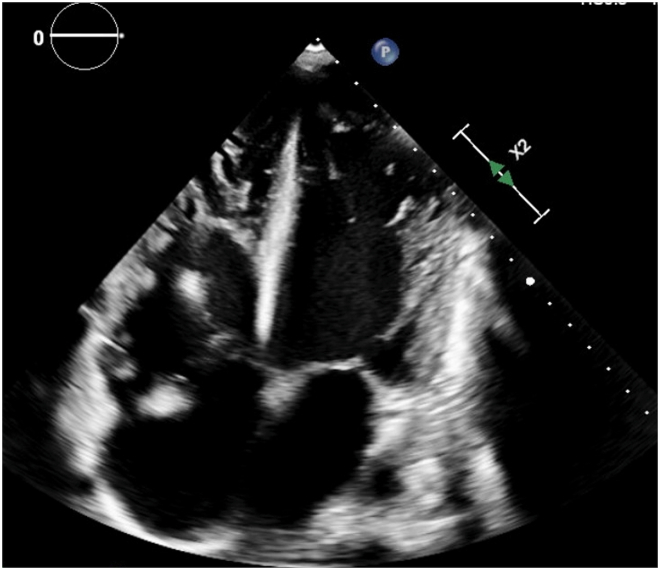
Initial Transthoracic Echocardiogram: Apical 4-Chamber View

On admission, laboratory evaluation revealed a B-type natriuretic peptide level of 4,674 pg/mL. High-sensitivity troponin I levels peaked at 6,580 pg/mL. Arterial blood gas demonstrated a lactate of 9.2 mmol/L and a pH of 7.11. Additional laboratory findings were notable for thrombocytopenia with a platelet count of 83 K/μL, mildly prolonged prothrombin time/international normalized ratio, and hypofibrinogenemia with a fibrinogen level of 51 mg/dL. Liver function tests showed mild transaminitis and hyperbilirubinemia. The initial ferritin, transferrin saturation, and hemoglobin were 15,710 ng/mL, 116%, and 13.8 g/dL, respectively. Transthoracic echocardiography (Figures 2 and 3) revealed severe biventricular systolic dysfunction, with a left ventricular ejection fraction of <20% and moderate left ventricular dilation (Videos 1 and 2), severe tricuspid regurgitation (Video 3), and bilateral ventricular thrombi (Figure 2). Computed tomography pulmonary angiography revealed bilateral ground-glass opacities suggestive of pulmonary edema, without evidence of pulmonary embolism. The visualized liver was normal in appearance. Coronary angiography was normal. Right heart catheterization demonstrated a reduced cardiac index of 1.24 L/min/m^2^, elevated right atrial pressure of 26 mm Hg, mean pulmonary artery pressure of 33 mm Hg, and pulmonary capillary wedge pressure of 19 mm Hg.

## Management

A temporary transvenous pacemaker was placed to address the patient's CHB. Additionally, a central venous cooling catheter was inserted to support targeted temperature management after her cardiac arrest. For her biventricular cardiogenic shock, she received intravenous furosemide and was initiated on inotropic and vasopressor support with dobutamine and epinephrine. Given the severity of her illness, she was transferred to a tertiary center for higher level of care. Anticoagulation for her biventricular thrombi was initially withheld given worsening coagulopathy in the context of thrombocytopenia.

**Figure 4:**
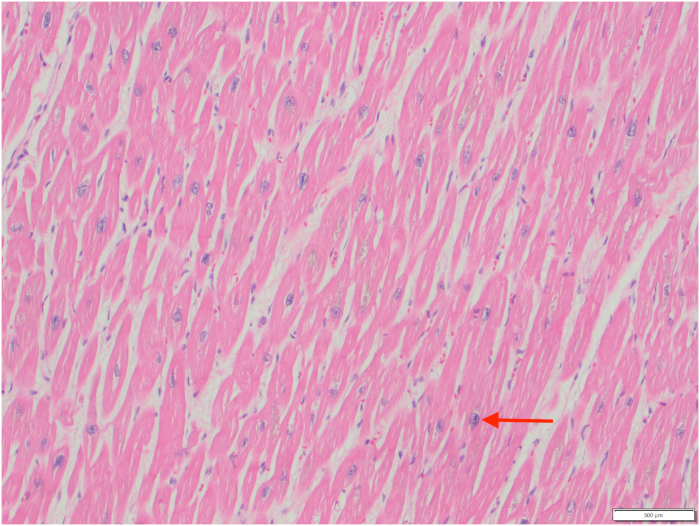
Endomyocardial Biopsy With Nonspecific Granular Pigment Deposition (Arrow)

**Figure 5:**
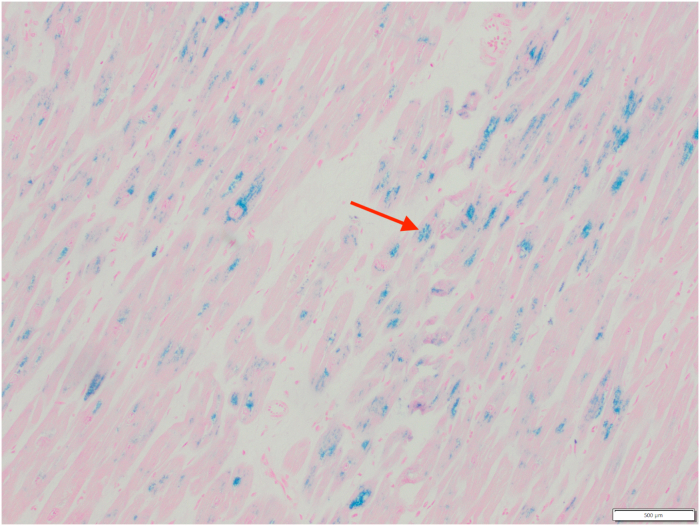
Endomyocardial Biopsy With Prussian Blue Staining Confirming Iron Deposition Within Cardiac Myocytes (Arrow)

**Figure 6:**
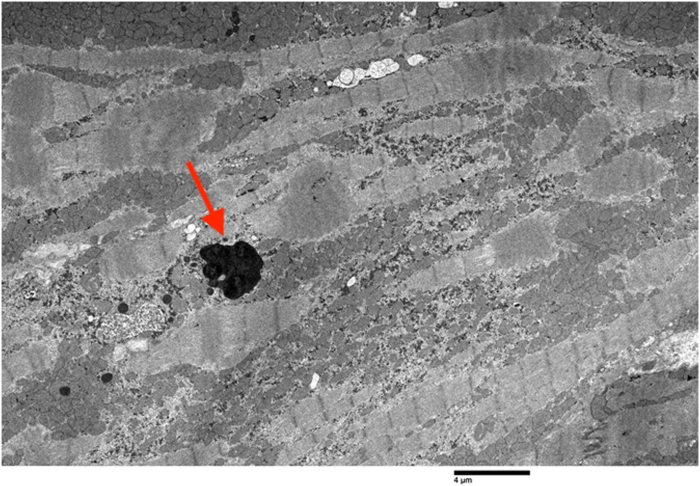
Electron Microscopy of the Endomyocardial Biopsy Demonstrating a Cluster of Iron-Rich Deposits Within Cardiac Myocytes (Arrow)

Upon arrival at the tertiary care center, she was admitted to the cardiovascular intensive care unit. With supportive measures, she progressively showed signs of stabilization. However, she unfortunately experienced a serious complication in the form of a large cerebrovascular accident, which was treated with mechanical thrombectomy. Given a lack of clinical improvement, her markedly abnormal iron studies from earlier in her hospital course were re-evaluated 4 weeks after admission, prompting endomyocardial biopsy and concurrent placement of a cardiac resynchronization therapy defibrillator device. Analysis of the biopsy demonstrated extensive iron deposition within cardiac myocytes without evidence of fibrosis or amyloid (Figure 4, Figure 5, Figure 6), confirming the diagnosis of an iron overload disorder. In response to these histological findings, a targeted hereditary hemochromatosis (HH) subtype panel was sent (PreventionGenetics),with identification of homozygous G320V mutation of the hemojuvelin (*HJV*) gene, consistent with juvenile hemochromatosis (JH) type 2A. The hematology team initiated dual-agent iron chelation therapy with intravenous deferoxamine and oral deferiprone.

## Outcome and Follow-Up

**Figure 7:**
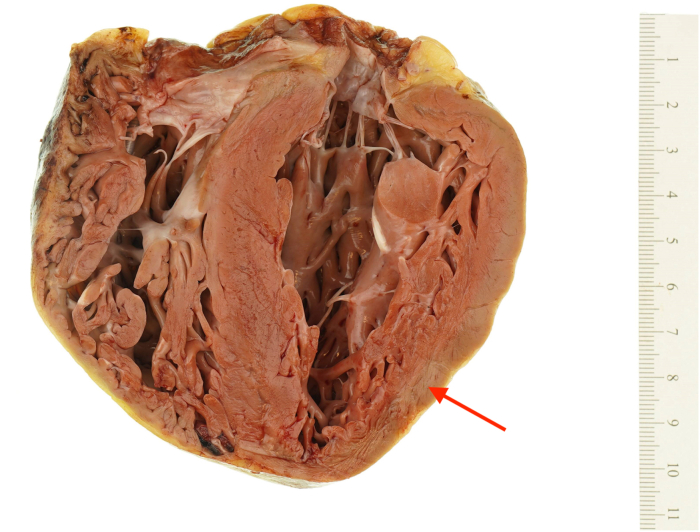
Explanted Heart Demonstrating Myocardial Hypertrophy and Scant Patchy Fibrosis (Arrow)

Over the next 2 months, the patient's hepatic and renal function recovered, the ventricular thrombi resolved with anticoagulation, her neurologic deficits improved, and she was eventually discharged. Despite optimization with guideline-directed medical therapy and normalization of her ferritin level, attempts at weaning her inotropic support were not successful, and she was deemed inotrope-dependent. She was identified as a transplant candidate and underwent successful isolated orthotopic heart transplantation 8 months after her initial presentation (Figure 7).

## Discussion

An endocrinology-focused description of this case was previously reported by Zinecker et al.^1^ In the present report, we describe the cardiovascular perspective and emphasize the underlying pathophysiology and clinical manifestations of JH as they pertain to the cardiovascular system.

HH is an autosomal recessive condition caused by genetic mutations that interfere with the absorption and regulation of iron in the human body.^2^ While cardiac infiltration is highlighted in this case, systemic involvement of the liver, pancreas, thyroid, connective tissue, and reproductive system is frequently observed later in the disease course, with ambiguous symptoms such as arthralgias, fatigue, weakness, and sexual dysfunction often seen upon initial presentation.^2^ States of iron overload have been associated with a variety of cardiovascular manifestations, several of which are of particular relevance to this case, including iron overload cardiomyopathy, conduction abnormalities, and tachyarrhythmias.^3^

The factors that contribute to either a restrictive or dilated phenotypic expression of iron overload cardiomyopathy are multifactorial, with the mechanism of iron overload, stage of disease progression, and interaction between an individual's genetics and immunoinflammatory factors all playing a role in which phenotype is observed clinically.^3^ While various genetic mutations have been implicated in the pathogenesis of HH, a homozygous C282Y mutation of the homeostatic iron regulator (*HFE*) gene is the most common.^4^ Despite estimates suggesting that a homozygous C282Y mutation in the *HFE* gene is present in roughly 1 in 200 people of Northern European descent, low penetrance limits clinically significant disease in most individuals.^4^ C282Y *HFE* mutations modestly diminish the ability of hepcidin to detect elevated serum iron levels, resulting in gradual, systemic iron deposition, with symptoms typically observed between the fourth and sixth decades of life.^4^ In contrast, cases of JH often present dramatically in patients younger than age 30 with rapidly progressive disease attributed to higher mutation penetrance and a profound deficiency of hepcidin.^4^ Recent investigations of genomic databases estimate that the HJV mutations described in this case affect only 1 in 4.8 million people globally.^5^

The *HJV* gene encodes a glycosylphosphatidylinositol anchor-linked protein involved in a signaling pathway that promotes the transcription of hepcidin through an incompletely understood mechanism.^6^ Homozygous mutation of *HJV* results in near-absence of hepcidin and a lack of negative feedback on ferroportin, the primary iron-exporting channel of enterocytes and macrophages.^4^^,^^6^ As transferrin rapidly becomes saturated with iron, substantial quantities of toxic, non–transferrin bound iron (NTBI) are deposited in tissue, leading to oxidative damage.^4^^,^^6^ Individuals with *HJV*-associated JH are more likely to experience disease that predominantly affects the cardiovascular system and the hypothalamic-pituitary-gonadal axis owing to an abundance of L-type calcium channels found in these tissues, which ferrous (Fe^2+^) NTBI readily enters in unregulated fashion.^4^^,^^6^ This is in contrast to classic, *HFE*-associated HH, where modest hepcidin deficiency results in slower, transferrin-mediated hepatic iron deposition before extrahepatic injury develops.^4^

Iron removal strategies such as phlebotomy, chelation, and erythrocytapheresis are mainstays of therapy, while novel hepcidin-mimetics such as rusfertide are currently being investigated.^4^ Despite being unsuccessful in this case, damage to cardiac tissue can be reversed to an extent via chelation, with associated recovery of ejection fraction in certain individuals otherwise destined for cardiac transplantation.^7^

We highlight several important teaching points from this case. Although this patient carried a prior diagnosis of idiopathic hypogonadism and was found to be notably tanned, to our knowledge, iron studies that could have explained the nature of her presentation had not been obtained before admission. Her markedly elevated ferritin level was initially misinterpreted by cardiovascular critical care teams and mistakenly attributed to a post–cardiac arrest inflammatory state, reflecting hyperferritinemia as a nonspecific acute-phase reactant. In reality, the profoundly elevated transferrin saturation reflected her true underlying pathology and was essential in leading care teams to the eventual diagnosis of JH. It was only after this abnormality was re-evaluated approximately 4 weeks after her presentation did concerns for hemochromatosis arise, prompting eventual endomyocardial biopsy. Importantly, descriptions of CHB secondary to HH are exceedingly rare, with no more than 3 instances described in the literature. The rarity of this presenting feature, coupled with early misinterpretation of her laboratory findings, likely contributed to diagnostic delay.Although CHB in this setting is associated with high mortality, clinicians should recognize that heart failure and ventricular arrhythmias are more common cardiac manifestations of HH. Therefore, clinicians must maintain a high index of suspicion for this disease in patients presenting with otherwise unexplained cardiomyopathy or conduction abnormalities.

## Conclusions

Rare but life-threatening presentations of *HJV*-associated JH are characterized by fulminant heart failure in the second and third decades of life, in contrast to traditional *HFE*-associated HH. This highly penetrant mutation leads to accelerated rates of iron bioaccumulation with a propensity for deposition in cardiac tissue and is associated with high rates of mortality owing to diagnosis often occurring at advanced stages of disease.

## Funding Support and Author Disclosures

The authors have reported that they have no relationships relevant to the contents of this paper to disclose.

## Appendix

Video 1 Initial Transthoracic Echocardiogram: Parasternal Long-Axis and Short-Axis Views Displaying Severe Biventricular Systolic Dysfunction

Video 2 Initial Transthoracic Echocardiogram: Apical 4-Chamber View Highlighting Severe Biventricular Systolic Dysfunction

Video 3 Initial Transthoracic Echocardiogram: Apical 4-Chamber View With Doppler Demonstrating Severe Tricuspid Regurgitation

## References

[1] Zinecker K.A., Sliwinska A., Wilson L.M., Zahr R. (2024). Juvenile hemochromatosis connecting cardiac arrest and hypogonadotropic hypogonadism in a young woman. JCEM Case Rep.

[2] Crownover B.K., Covey C.J. (2013). Hereditary hemochromatosis. Am Fam Physician.

[3] Kremastinos D.T., Farmakis D. (2011). Iron overload cardiomyopathy in clinical practice. Circulation.

[4] Girelli D., Marchi G., Busti F. (2024). Diagnosis and management of hereditary hemochromatosis: lifestyle modification, phlebotomy, and blood donation. Hematol Am Soc Hematol Educ Program.

[5] Wallace D.F., Subramaniam V.N. (2016). The global prevalence of HFE and non-HFE hemochromatosis estimated from analysis of next-generation sequencing data. Genet Med.

[6] Galy B., Conrad M., Muckenthaler M. (2024). Mechanisms controlling cellular and systemic iron homeostasis. Nat Rev Mol Cell Biol.

[7] Cooray S.D., Heerasing N.M., Selkrig L.A. (2018). Reversal of end-stage heart failure in juvenile hemochromatosis with iron chelation therapy: a case report. J Med Case Rep.

